# A45 FIBER-FREE DIET REDUCES BACTEROIDES ABUNDANCE AND PREVENTS MUCUS DEGRADATION IN MICE COLONIZED WITH MICROBIOTA FROM PATIENTS WITH GENERALIZED ANXIETY DISORDER

**DOI:** 10.1093/jcag/gwac036.045

**Published:** 2023-03-07

**Authors:** V Rabbia, G De Palma, P Bercik, J Lu, E Verdu, S Collins, M Surette

**Affiliations:** Medicine, McMaster University, Hamilton, Canada

## Abstract

**Background:**

Generalized anxiety disorder (GAD) is a debilitating condition with a lifetime prevalence of 4-7% worldwide. We have previously found that compared to healthy controls, GAD patients had lower reported fiber intake, increased gastrointestinal symptoms; and enrichment of *Bacteroides* genus as well as carbohydrate metabolism pathways (as determined by PICRUSt2, correlated to *Bacteroides* abundance). *Bacteroides* are known for its ability to degrade a wide variety of host polysaccharides, such as the intestinal mucus, which could lead to local and systemic inflammation. In this regard, GAD patients had higher C-reactive protein (CRP) compared to healthy controls (p=0.049).

**Purpose:**

To investigate whether a fiber-free diet could decrease *Bacteroides* abundance and prevent damage of the mucus layer reducing anxiety-behavior in mice with GAD microbiota.

**Method:**

Two germ-free NIH Swiss mouse breeding pairs were colonized with GAD microbiota using patients’ stool samples and kept on either fiber-free or 10 % inulin (fiber) diet. Offspring were weaned at week 3 and psychometric tests were performed at 10 weeks of age. After sacrifice, samples for histology (mucus layer thickness determination), blood (CRP ELISA determination) and stool (Illumina 16S rRNA gene sequencing) were collected. The microbiota data was analyzed following the pipelines of dada2 and by mean comparisons, correlation, AncomBC using R software (v.1.2.1335). Multiple comparison results were corrected allowing 5% of FDR.

**Result(s):**

Beta diversity analysis showed that parent and offspring’s (n=24 fiber-supplemented and n=14 fiber-free groups) microbiota was similar to the GAD donor. The most differentially abundant bacterial taxon was *Bacteroides uniformis*, which was decreased in the fiber-free group (p.adj= 0.003). Furthermore, fiber-free diet reduced the overall *Bacteroides* abundance by half compared to the donor and fiber-supplemented mice group. This led to a thickening (p.adj=0.027) of the mucus layer, increasing from 30 µm (fiber-supplemented diet) to 60 µm in the fiber-free diet group. *B. uniformis* was negatively correlated to the mucus layer thickness (R= -0.81; p.adj=0.26), although not statistically significant, likely due to a low n number (n=4). We only found a statistical trend for higher CRP levels and anxiety-like behavior in the fiber-supplemented group. This might be because fiber supplementation has several beneficial effects that can counteract the proposed increase in anxiety-like behavior from*Bacteroides*. Despite that, we found a significant correlation between *B. uniformis* and time mice spent in the dark (indicative of anxiety-like behavior) in the light preference test.

**Conclusion(s):**

Our data suggests that *Bacteroides *abundance, specifically *Bacteroides uniformis, *contributes to the degradation of the mucus layer and potentially triggers low grade gut inflammation and anxiety-like behavior.

**Please acknowledge all funding agencies by checking the applicable boxes below:**

CIHR

**Disclosure of Interest:**

None Declared

